# Systematic Review and Meta-Analysis of Clinical Severity and Population-Specific Outcomes of Human Metapneumovirus Infection

**DOI:** 10.1093/ofid/ofag634

**Published:** 2026-10-08

**Authors:** Yu Miao, Shangchen Song, Zachary J Madewell, Zhumengmeng Jin, Yumiao Lei, Evelyn Shi, Yixuan Zeng, Mingjing Liu, Ira M Longini, Yang Yang

**Affiliations:** Department of Statistics, Franklin College of Arts and Sciences, University of Georgia, Athens, Georgia, USA; Center for Biologics Evaluation and Research, U.S. Food and Drug Administration, Silver Spring, Maryland, USA; Department of Biostatistics, University of Florida, Gainesville, Florida, USA; Silver Spring, Maryland, USA; Department of Statistics, Franklin College of Arts and Sciences, University of Georgia, Athens, Georgia, USA; Department of Biostatistics, University of Florida, Gainesville, Florida, USA; Department of Statistics, Franklin College of Arts and Sciences, University of Georgia, Athens, Georgia, USA; Department of Epidemiology and Health Statistics, Xiangya School of Public Health, Central South University, Changsha, China; Department of Biostatistics, University of Florida, Gainesville, Florida, USA; Department of Statistics, Franklin College of Arts and Sciences, University of Georgia, Athens, Georgia, USA

## Abstract

**Background:**

Human metapneumovirus (HMPV) is a prevalent respiratory pathogen, but comprehensive estimates of severe clinical outcomes across populations are lacking. We synthesized the proportion of severe outcomes among people with laboratory-confirmed HMPV infection.

**Method:**

We searched PubMed, Embase, Web of Science, Scopus, Cochrane Library, medRxiv, bioRxiv, and Google Scholar from inception to 20 January 2026, without language restrictions. Observational studies reporting severe outcomes (hospitalization, intensive care unit [ICU] admission, respiratory support, mechanical ventilation, mortality) in laboratory-confirmed HMPV infection were eligible; studies enrolling only people who are immunocompromised without comparators were excluded. Two reviewers independently extracted data and assessed quality using Joanna Briggs Institute checklists. Pooled proportions were estimated using random-effects generalized linear mixed models with logit transformation. Subgroup analyses examined healthcare setting and age.

**Results:**

Ninety-four studies (∼26 000 people who are HMPV positive) were included. Pooled proportions were 48% (95% CI, 20%–78%; 8 studies, n = 1445) for hospitalization; 12% (95% CI, 8%–16%; 26 studies, n = 14 522; Egger *P* = .03) for ICU admission; 40% (95% CI, 26%–56%; 18 studies, n = 4328) for respiratory support; 8% (95% CI, 5%–13%; 16 studies, n = 5543) for mechanical ventilation; and 3% (95% CI, 2%–5%; 29 studies, n = 13 657; I^2^ = 78.5%) for in-hospital mortality. Mortality was highest among adults (6%).

**Conclusions:**

Human metapneumovirus causes substantial hospitalization, ICU admission, and mortality, supporting strengthened surveillance, integration into respiratory virus prevention strategies, and accelerated vaccine development.

Human metapneumovirus (HMPV) is recognized as a leading cause of acute respiratory tract infections globally, frequently ranking second only to respiratory syncytial virus (RSV) among children younger than 5 years in outpatient and hospital settings [1–5]. With the widespread adoption of nucleic acid amplification tests (NAATs), contemporary studies have shown that hospitalization and adverse clinical outcomes among older adults with HMPV may be comparable to those associated with RSV or influenza [6–8]. Yet HMPV currently lacks both a licensed vaccine and a targeted antiviral therapy, making comprehensive severity estimates essential for guiding prevention priorities and therapeutic development. Together, these findings identify HMPV as an important, though often underrecognized, seasonal contributor, alongside RSV, influenza, and Severe acute respiratory syndrome coronavirus 2 (SARS-CoV-2) [7, 8]. Moreover, the frequent co-occurrence of HMPV with other pathogens that share similar symptoms further complicates clinical diagnosis and accurate attribution of morbidity and mortality [8].

Despite growing evidence of HMPV's global impact across all ages, quantitative synthesis of severe outcomes remains limited. Previous systematic reviews have primarily focused on incidence, prevalence, or global burden, largely in children [5, 9, 10]. To our knowledge, no study has systematically assessed pooled key severity outcomes, including hospitalization, intensive care unit (ICU) admission, need for respiratory support, mechanical ventilation, and mortality across diverse geographic settings and age groups. Robust pooled estimates of disease severity are essential for informing clinical risk stratification, hospital surge preparedness, resource allocation, and public health policy.

In this systematic review and meta-analysis, we aimed to estimate the proportions of people who are HMPV positive experiencing major severe clinical outcomes across age groups to inform priorities for surveillance, clinical management, and vaccine development.

## METHODS

This systematic review and meta-analysis were conducted according to the Preferred Reporting Items for Systematic Reviews and Meta-Analyses (PRISMA) 2020 guidelines [11].

### Search Strategy

We systematically searched PubMed (1946–present), Embase (1947–present), Web of Science (1900–present), Scopus (1970–present), and the Cochrane Library (1996–present) through 20 January 2026 without language restrictions. All search terms were in English. The search strategy combined 4 concept groups: (1) virus identification terms (HMPV), (2) severity outcome terms (mortality, hospitalization, ICU, mechanical ventilation), (3) study design terms (cohort, case–control, cross-sectional, prospective, retrospective), and (4) statistical analysis terms (odds ratio, risk ratio, hazard ratio, CI). The complete search strategy is provided in Supplementary Table 1. We also searched medRxiv and bioRxiv using a simplified query and screened the first 100 Google Scholar results for relevant studies. All retrieved records were imported into Zotero for reference management and automated deduplication, with any remaining duplicates resolved manually. Reference lists of included studies and relevant reviews were manually screened.

### Study Selection

Eligibility followed the PICOS framework. We included individuals of any age with laboratory-confirmed HMPV infection, primarily by reverse transcription polymerase chain reaction (RT-PCR) or NAATs; immunofluorescence or viral culture was accepted for earlier studies conducted before widespread NAAT/PCR use. Studies relying solely on clinical diagnosis were excluded, as were those focused exclusively on immunocompromised populations without a comparison group. Primary outcomes were hospitalization, ICU admission, respiratory support, mechanical ventilation, and in-hospital mortality. Secondary outcomes included author-defined severity indices, complications, and reported risk factors when available; these outcomes were extracted descriptively because definitions and reporting were not sufficiently consistent for quantitative synthesis. Eligible designs were primary observational studies providing extractable quantitative data, including cohort, case–control, cross-sectional, and surveillance designs, which provided extractable quantitative data on HMPV-specific severe outcomes. Other primary study designs, including case series or randomized trial reports, were eligible if HMPV-specific clinical outcome data could be extracted descriptively; trial reports were not used to estimate intervention effects. Titles and abstracts were screened independently by reviewer pairs, with disagreements resolved by consensus. Full texts of potentially eligible studies were assessed against PICOS criteria.

### Data Extraction and Quality Assessment

Data were extracted independently by reviewer pairs using standardized forms with a comprehensive data dictionary. Prior to full extraction, 4 experienced reviewers (Y. M., Y. Y., S. S., Z. J. M.) jointly piloted the process on 5 studies to harmonize interpretation. For each study, reviewers extracted study-level characteristics (design, setting, demographics, diagnostic methods, sample sizes), clinical outcomes stratified by population subgroups, quantitative clinical indicators, and risk factors for severe outcomes. Risk factors were extracted as reported by the original study authors, including the risk-factor variable, severe outcome assessed, population or subgroup, effect measure when available, and whether the association was based on univariable or multivariable analysis. Because risk factors were defined and modeled inconsistently across studies, they were summarized descriptively and were not pooled quantitatively. For studies reporting multiple populations, separate data entries were created for subgroups and for the overall population. Study settings were categorized based on the primary study recruitment site: hospital-based (studies conducted at hospital facilities, including admitted inpatients and people presenting to hospital emergency departments), ICU-based (people admitted to ICUs), emergency department/clinic-based (freestanding emergency departments or community-based outpatient clinics not part of hospital systems), community-based (population surveillance or community screening), or mixed (studies recruiting from multiple settings without disaggregated reporting). Discrepancies were resolved through discussion, with consensus data entered into final workbooks.

The quality of all included papers was assessed based on the appropriate Joanna Briggs Institute (JBI) critical appraisal checklist for each study design [12]. Each question was rated as yes, no, or unclear. For each study, the JBI percentage score was calculated as the number of applicable checklist items rated “yes” divided by the total number of applicable items, multiplied by 100. Studies with scores of 70% or higher were classified as higher quality, and studies with scores below 70% were classified as lower quality. Unclear responses were treated as not satisfied (ie, penalizing insufficient reporting). The ≥70% threshold was used pragmatically to define higher-quality studies for planned sensitivity analyses and was not treated as a formally validated risk-of-bias cutoff. The JBI percentage summary was not used for meta-analytic weighting, and no studies were excluded from the primary analyses on the basis of this summary. Joanna Briggs Institute checklists were selected because this review synthesized observational evidence reporting proportions of severe clinical outcomes, rather than intervention effects. Study-level JBI critical appraisal results for all 94 included studies are provided in Supplementary Table 4.

### Data Synthesis and Analysis

We defined 5 severity outcomes using a standardized operational framework informed by WHO clinical guidance for severe acute respiratory infection and CDC respiratory virus hospitalization surveillance frameworks [12, 13]. To ensure consistency across included studies, the denominator for each outcome was explicitly defined. Hospitalization was defined as the proportion of people who are HMPV positive seeking healthcare (inclusive of outpatient and emergency department settings) who were admitted to inpatient care. Intensive care unit admission was defined as the proportion of people who are hospitalized with admission to or transfer to an ICU. Supplemental respiratory support was defined as supplemental oxygen or oxygen-delivery support not classified as ventilatory support, including nasal cannula, face mask oxygen, and high-flow nasal cannula when reported. CPAP and BiPAP were classified as noninvasive ventilation when reported separately and were therefore included under mechanical ventilation/noninvasive ventilation rather than under supplemental respiratory support. Mechanical ventilation included invasive ventilation via endotracheal or tracheostomy tube and/or noninvasive ventilation when reported together. When primary studies reported only a composite respiratory-support category without separating oxygen therapy from CPAP, BiPAP, or other ventilation modalities, data were extracted according to the authors’ reported definition and interpreted cautiously. In-hospital mortality was defined as death from any cause during the index hospitalization, representing a proportion of people who are hospitalized.

For each outcome, only studies reporting the relevant data were included in the corresponding meta-analysis; thus, the number of contributing studies varied across outcomes. Mixed-setting studies were analyzed as a separate subgroup when included and were excluded from analyses requiring setting-specific denominators, such as hospitalization, when the recruitment setting or denominator could not be interpreted consistently. Age categories were harmonized into 3 groups (pediatric, <18 years; adults, ≥18 years; and mixed). Mixed-age studies were retained when HMPV-specific outcome numerators and denominators were extractable, but they were analyzed as a separate mixed-age category and were not assigned to either the pediatric or adult subgroup. Pooled proportions were estimated using random-effects generalized linear mixed models (GLMMs) with a logit link, specifying study as a random intercept. Between-study variance (τ^2^) was estimated by maximum likelihood, and Hartung–Knapp–Sidik–Jonkman adjustment was applied to CIs of the pooled estimates to account for uncertainty in the heterogeneity estimate. CIs were calculated on the logit scale and back transformed to the proportion scale. Heterogeneity was assessed using τ^2^ and I^2^. Prespecified subgroup analyses were conducted by study setting and age group. Hospitalization analyses were restricted to noncommunity settings. Small-study effects and potential publication bias were evaluated using funnel plots and Egger's regression test for each major outcome [13]. For Egger's regression, study-level proportions were logit transformed prior to analysis, and the regression was weighted by the inverse variance of these transformed estimates. Sensitivity analyses excluded poor-quality studies (JBI score < 70%). All statistical analyses were conducted using R version 4.2.3 (R Core team, 2023) with the meta package [14].

Models were rerun after excluding studies rated as lower quality (scoring below 70% on the JBI critical appraisal checklist). Differences in pooled proportions, 95% CIs, and heterogeneity metrics between the full dataset and the high-quality subset were summarized to assess consistency of results.

In addition to pooled analyses, we systematically summarized clinical indicators frequently reported but unsuitable for meta-analysis (eg, length of hospital stay and ICU stay). These outcomes were summarized descriptively because they showed substantial heterogeneity in definitions, measurement timing, summary measures, denominator clarity, subgroup reporting, and completeness of reporting across studies.

## RESULTS

### Study Selection and Characteristics

Our search yielded 840 records across all databases. One additional record was identified through citation searching. After removing 287 duplicates, 554 unique records were screened by title and abstract, of which 344 were excluded. Following full-text review of 210 reports, 116 were excluded: HMPV-specific data not extractable (n = 70), wrong focus (n = 19), exclusively immunocompromised populations (n = 16), ineligible publication or study design (n = 6), and other reasons, including duplicate populations (n = 5). In total, 94 studies [3, 6, 7, 15–104], comprising approximately 26 000 people who are HMPV positive (Supplementary Table 2), met inclusion criteria (Figure 1).

**Figure 1.**
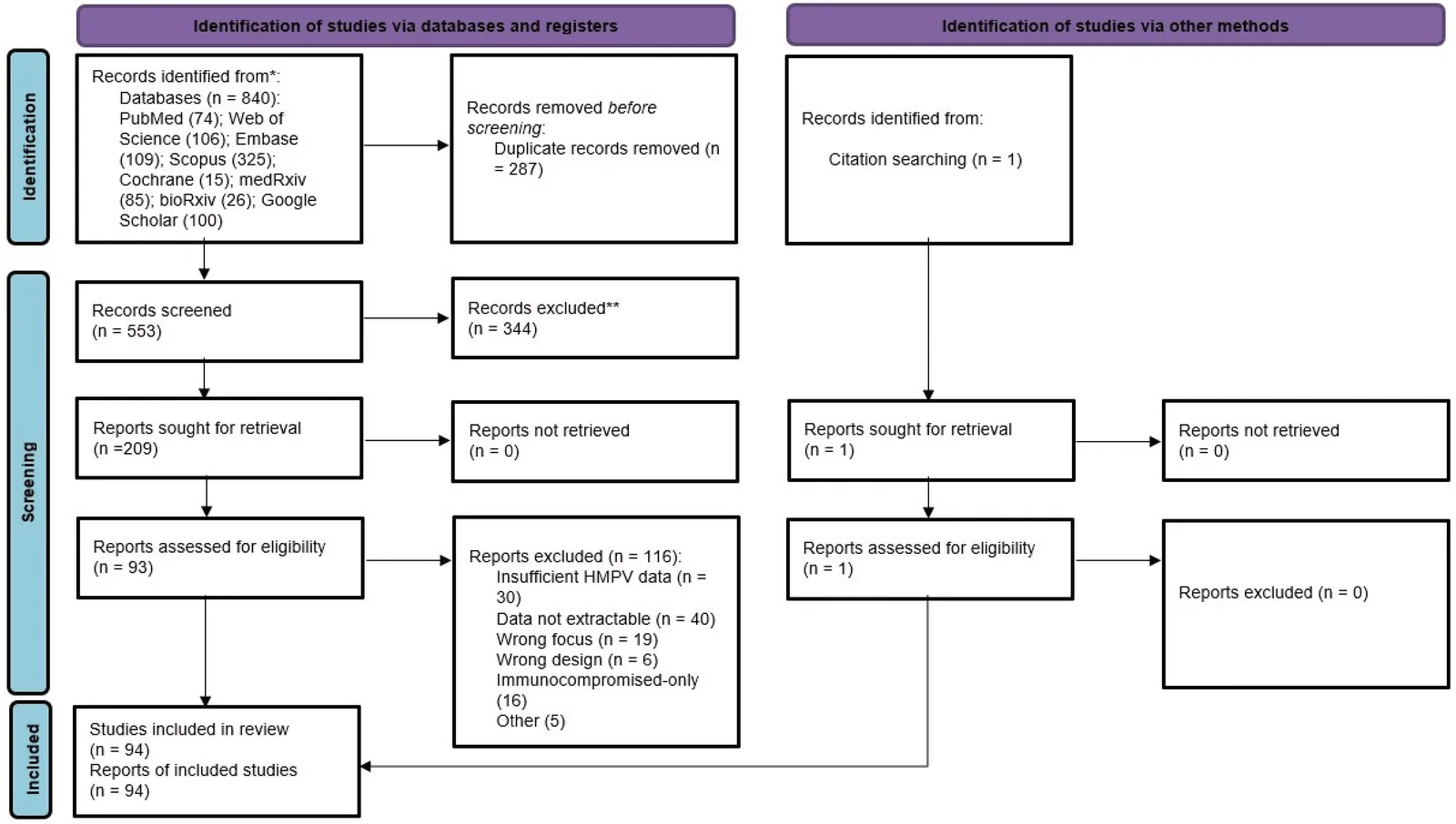
Preferred Reporting Items for Systematic Reviews and Meta-Analyses (PRISMA) 2020 flow diagram for the systematic review of human metapneumovirus clinical severity (search date: 20 January 2026).

Studies were geographically diverse, spanning high-, middle-, and low-income countries from North America, Europe, Asia, Africa, and Latin America, with several multinational cohorts. Cohort studies predominated (70 of 94 [74.5%]; prospective or retrospective), followed by case–control (10 [10.6%]) and cross-sectional (7 [7.4%]) designs. The remaining studies used surveillance, case series, or randomized controlled trial frameworks (7 [7.4%]). Most were hospital-based (61 [64.9%]), with mixed settings (16 [17.0%]), community-based surveillance (7 [7.4%]), ED/clinic-based (6 [6.4%]), and ICU-based cohorts (4 [4.3%]). Diagnostic confirmation was primarily by PCR/NAAT (83 [88.3%]), followed by immunofluorescence (6 [6.4%]) and other methods (5 [5.3%]). Study periods ranged from 1976 to 2026 (Table 1). For quantitative synthesis, data were available from 8 studies on hospitalization, 26 on ICU admission, 18 on respiratory support, 15 on mechanical ventilation, and 29 on in-hospital mortality. Other outcomes, such as hospital length of stay (LOS), were summarized descriptively.

**Table 1.** Characteristics of Included Studies on HMPV-Associated Severe Outcomes (N = 94)

Characteristic	Category	No. of Studies (%)
Study design	Cohort (prospective/retrospective)	70 (74.5%)
	Case–control	10 (10.6%)
	Cross-sectional	7 (7.4%)
	Other (surveillance, case series, randomized trial report)	7 (7.4%)
Study setting	Inpatient	61 (64.9%)
	Mixed	16 (17.0%)
	Community-based	7 (7.4%)
	Outpatient/emergency department	6 (6.4%)
	ICU-based	4 (4.3%)
Population focus	Pediatric	58 (61.7%)
	Adult	24 (25.5%)
	Mixed age	12 (12.8%)
Diagnostic method	PCR/NAAT (generic)	74 (78.7%)
	Multiplex PCR	9 (9.6%)
	Immunofluorescence assay	6 (6.4%)
	Other/unspecified	5 (5.3%)
Country income levels^a^	High income	52 (55.3%)
	Upper–middle income	25 (26.6%)
	Lower–middle income	10 (10.6%)
	Mixed/multinational	4 (4.3%)
	Low income	3 (3.2%)
Study period (start year)	Before 2010^b^	24 (25.5%)
	2010–2019	64 (68.1%)
	2020–present	6 (6.4%)

Abbreviations: HMPV, human metapneumovirus; ICU, intensive care unit; NAAT, nucleic acid amplification test; PCR, polymerase chain reaction.

^a^World Bank income classification based on the year of study conduct.

^b^Before 2010 was used to distinguish earlier studies conducted before widespread adoption of multiplex NAAT/PCR testing for respiratory virus diagnosis.

Complications and risk factors were inconsistently defined and reported across studies and therefore were not meta-analyzed; no standardized cross-study summary was attempted.

### Pooled Estimates of Severe Outcomes

#### Hospitalization

For hospitalization outcomes, we restricted analysis to emergency department/clinic-based and hospital-based studies (including people presenting to hospital emergency departments and outpatient clinics), excluding community-based surveillance studies or mixed settings due to fundamental differences in population denominators and case ascertainment. Hospitalization proportions ranged from 0% to 89% across 8 individual studies from either emergency department or hospital-based settings. Across all included studies (n = 1445 people who are HMPV positive), the overall pooled proportion requiring hospitalization was 48% (95% CI, 20%–78%) (I^2^ = 97.7%; τ^2^ = 3.37) (Supplementary Figure 1). Three emergency department/clinic-based studies showed a pooled hospitalization rate of 43% (95% CI, 14%–77%), while 5 hospital-based studies showed 49% (95% CI, 11%–89%), with no significant difference between healthcare settings (χ^2^ = 0.04; df = 1; *P* = .84).

When stratified by age, the pooled rate was 66% (95% CI, 46%–82%) for 5 pediatric studies and 46% (95% CI, 9%–87%) for 2 mixed-age studies. The 5 pediatric studies enrolled children across different age ranges: 1 month–5 years, 0–5 years, 0–18 years, 0–3 years, and 1 month–18 years. Individual pediatric hospitalization proportions ranged from 33% to 89%, with higher estimates generally observed in studies focused on infants and young children. There was no significant difference among age groups (χ^2^ = 0.55; df = 2; *P* = .76) (Supplementary Figure 2). Because hospitalization outcomes were derived from selected healthcare-seeking or tested populations, this estimate should not be interpreted as the hospitalization risk among all HMPV infections or all emergency department presentations.

#### Supplemental Respiratory Support

Across 18 studies (n = 4328), the pooled proportion requiring supplemental respiratory support was 40% (95% CI, 26%–56%; I^2^ = 97.4%). There was no statistically significant difference in pooled supplemental respiratory-support proportions between settings (χ^2^ = 2.32; *P* = .31), although heterogeneity remained substantial overall (I^2^ = 97.4%): 45% across 14 hospital-based studies, 32% in 1 community-based study, and 23% across 3 mixed-setting studies (Supplementary Figures 3–4).

### Intensive Care Unit Admission

Across 26 studies (n = 14 522), the pooled ICU admission proportion was 12% (95% CI, 8%–16%; I^2^ = 96.8%), with individual study proportions ranging from 0.01% to 55% (Figure 2). Subgroup analysis revealed significant differences by setting (χ^2^ = 6.54; *P* = .04): 10% (95% CI, 7%–15%) across 23 hospital-based studies, 34% (95% CI, 15%–60%) across 2 mixed-setting studies, and 13% (95% CI, 6%–24%) in 1 other-setting study. No significant differences were found between age groups (χ^2^ = 0.78; *P* = .68): 10% (95% CI, 6%–17%) in pediatric patients (16 studies), 14% (95% CI, 9%–21%) in adults (9 studies), and 12% (95% CI, 6%–23%) in mixed-age groups (2 studies) (Supplementary Figure 5).

**Figure 2.**
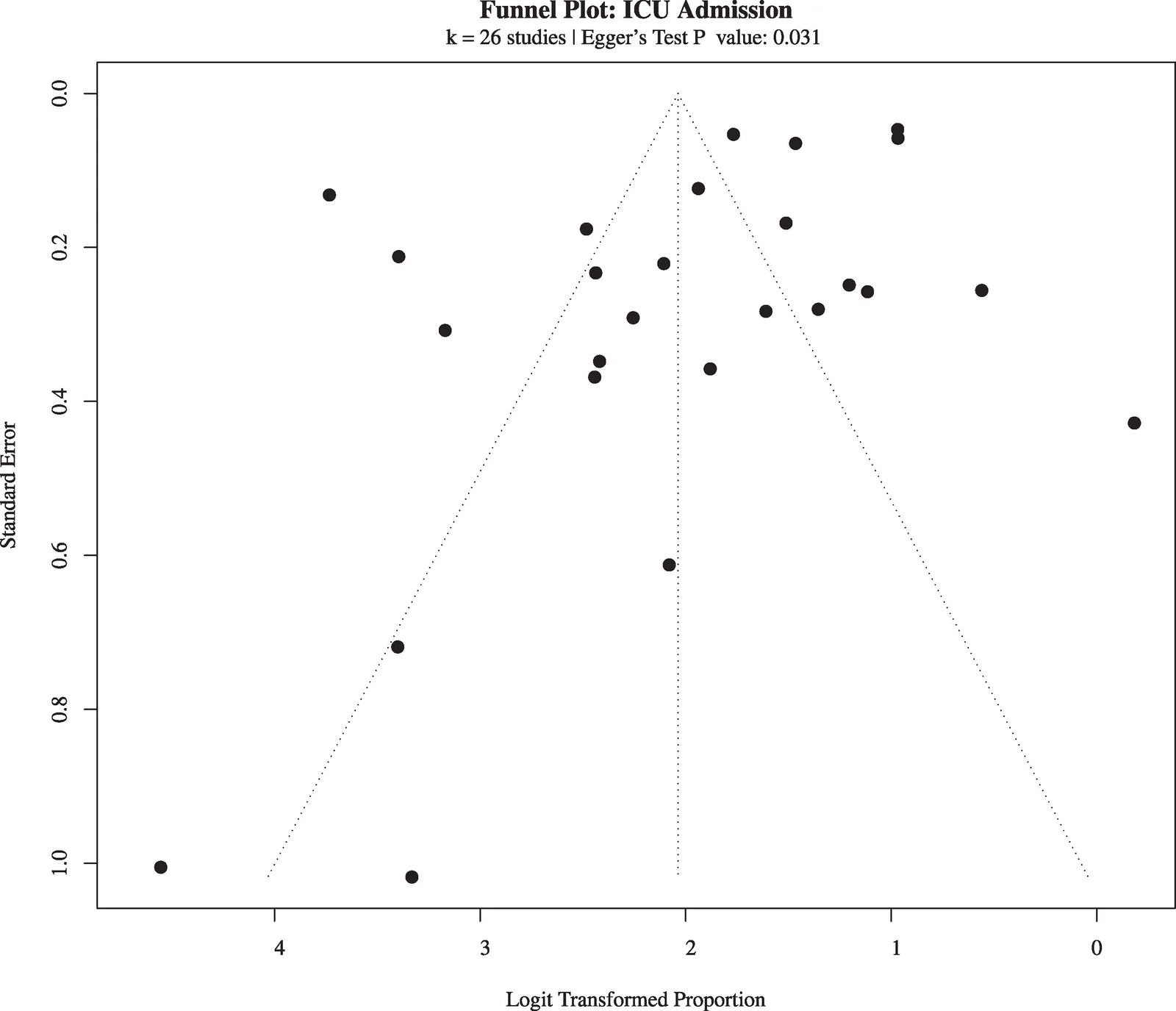
ICU admission among people with laboratory-confirmed HMPV infection, stratified by study setting. Squares indicate study-specific proportions, and horizontal lines indicate 95% CIs. Diamonds represent pooled random-effects estimates within each subgroup and overall (generalized linear mixed model [GLMM] with logit link). The horizontal bar indicates the 95% prediction interval for the overall pooled estimate. Abbreviations: CC, case–control; CI, confidence interval; Cross, cross-sectional; HIC, high-income country; HMPV, human metapneumovirus; ICU, intensive care unit; IF, immunofluorescence; JBI, Joanna Briggs Institute critical appraisal percentage; LMIC, lower–middle-income country; NAATs, nucleic acid amplification tests; PCR, polymerase chain reaction; Prosp, prospective; Retro, retrospective; Surv, surveillance; UMIC, upper–middle-income country.

### Mechanical Ventilation

Across 16 studies (n = 5543), the pooled proportion requiring mechanical ventilation was 8% (95% CI, 5%–13%; I^2^ = 94.0%). Substantial differences were noted by setting (χ^2^ = 62.45; *P* < .001): 6% (95% CI, 4%–9%) across 12 hospital-based studies, 20% (95% CI, 1%–72%) in 1 ICU-based study, and 15% (95% CI, 4%–44%) across 2 mixed-setting studies. No significant differences were observed across age groups: 6% (95% CI, 4%–11%; 10 studies) in pediatric patients, 11% (95% CI, 5%–20%; 4 studies) in adults, and 13% (95% CI, 2%–56%; 2 studies) in mixed-age populations (χ^2^ = 1.59; *P* = .45) (Supplementary Figures 6 and 7).

### In-Hospital Mortality

Across 29 studies (n = 13 657), pooled in-hospital mortality was 3% (95% CI, 2%–5%; I^2^ = 78.5%), with individual proportions ranging from 0% to 20% (Figure 3). Significant differences were observed by setting (χ^2^ = 11.12; df = 3; *P* = .01): 9% (95% CI, 4%–16%) in 1 community-based study, 3% (95% CI, 2%–5%) in 20 hospital-based studies, 20% (95% CI, 1%–72%) in 1 ICU-based study, and 3% (95% CI, 1%–6%) in 7 mixed-setting studies. Mortality was highest in adults (6%; 95% CI, 4%–8%; 10 studies), followed by pediatric patients (3%; 95% CI, 2%–4%; 13 studies) and mixed-age groups (2%; 95% CI, 1%–4%; 6 studies; χ^2^ = 12.54; *P* < .01) (Supplementary Figure 8).

**Figure 3.**
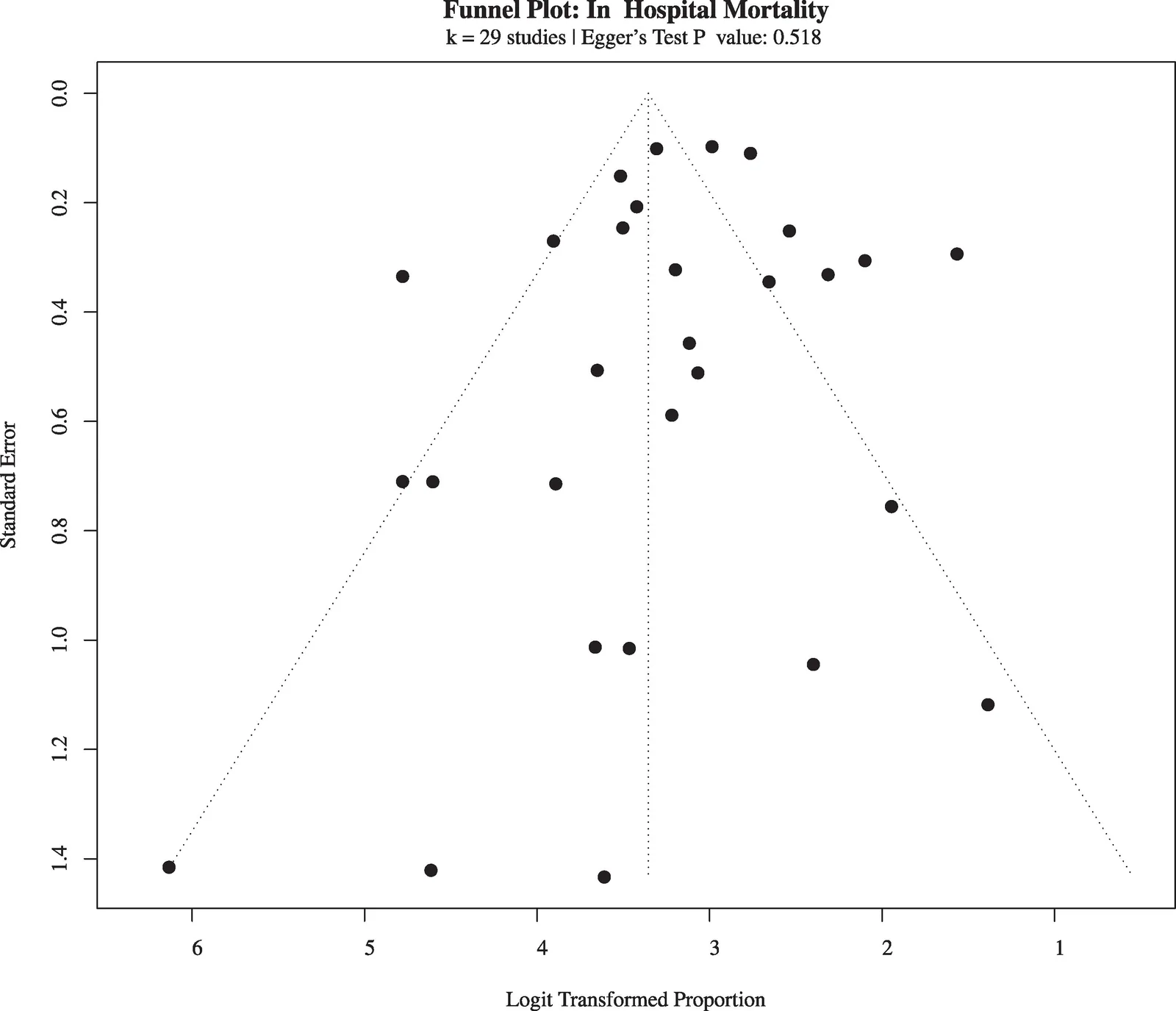
In-hospital mortality among people with laboratory-confirmed HMPV infection, stratified by study setting. Squares indicate study-specific proportions, and horizontal lines indicate 95% CIs. Diamonds represent pooled random-effects estimates within each subgroup and overall (GLMM with logit link). The horizontal bar indicates the 95% prediction interval for the overall pooled estimate. Abbreviations: CC, case–control; CI, confidence interval; Cross, cross-sectional; HIC, high-income country; HMPV, human metapneumovirus; ICU, intensive care unit; IF, immunofluorescence; JBI, Joanna Briggs Institute critical appraisal percentage; LIC, low-income country; LMIC, lower–middle-income country; NAATs, nucleic acid amplification tests; Prosp, prospective; RCT, randomized controlled trial; Retro, retrospective; UMIC, upper–middle-income country.

### Publication Bias and Sensitivity Analyses

Egger's tests indicated no significant small-study effects for in-hospital mortality (*P* = .52), mechanical ventilation (*P* = .95), supplemental respiratory support (*P* = .55), or hospitalization (*P* = .62) (Supplementary Figures 9–12). Significant small-study effects were observed for ICU admission (*P* = .03). Sensitivity analyses excluding studies with JBI critical appraisal percentages < 70% yielded consistent results across all outcomes (Table 2): In-hospital mortality was essentially unchanged (3.4% vs 3.5%, 1 study excluded), with modest shifts for ICU admission (11.5%–13.5%, 5 studies excluded), mechanical ventilation (7.9%–9.4%, 3 studies excluded), supplemental respiratory support (40.0%–41.3%, 3 studies excluded), and hospitalization (48.2%–39.6%, 2 studies excluded). CIs overlapped substantially between the full and higher-quality sensitivity analyses, and heterogeneity remained high (I^2^ ≥ 78.5% for all outcomes), suggesting that the pragmatic JBI classification was not a major driver of between-study variation.

**Table 2.** Full Dataset vs Higher-Quality Studies (JBI ≥ 70% for Sensitivity Analysis Only)

Outcome	All Eligible Studies			High Quality Studies				
	N	Estimate (95% CI)	I^2^%	N	Estimate (95% CI)	I^2^%	Excluded	Δ
ICU admission	26	0.115 (0.082–0.160)	96.8	21	0.135 (0.096–0.185)	95.6	5	+0.019
Mechanical ventilation	16	0.079 (0.048–0.128)	94.0	13	0.094(0.054–0.157)	95.0	3	+0.014
Supplemental respiratory support	18	0.400 (0.259–0.560)	97.4	15	0.413 (0.266–0.578)	97.7	3	+0.013
In-hospital mortality	29	0.034 (0.024–0.047)	78.5	28	0.035 (0.025–0.049)	78.5	1	+0.002
Hospitalization	8	0.482 (0.199–0.776)	97.7	6	0.396 (0.098–0.798)	98.2	2	−0.086

All = meta-analysis including all eligible studies; HQ = higher-quality studies for sensitivity-analysis purposes only (defined as ≥70% “Yes” ratings for applicable items on the JBI critical appraisal checklist; studies below this pragmatic threshold were excluded from HQ analyses and plots). The 70% threshold is not a validated JBI risk-of-bias cutoff and was not used for meta-analytic weighting. Estimates are pooled proportions (GLMM with logit link, sm = PLOGIT); I^2^ indicates heterogeneity. Δ = (HQ pooled estimate) − (all pooled estimate).

### Healthcare Utilization

Hospital LOS varied substantially across populations and settings (Supplementary Table 3). Although median hospital LOS showed substantial overlap between pediatric (range of medians, 1.5–24 days) and adult populations (range of medians, 3–10 days), children's hospital LOS appeared shorter than adults’. Across 8 studies, the median ICU LOS was generally 2–4 days (range, 1–7 days), with greater variability observed in some pediatric ICU cohorts.

## DISCUSSION

In this systematic review and meta-analysis of 94 studies [3, 6, 7, 15–104], we found that HMPV is a significant cause of morbidity and mortality comparable to RSV and influenza. Among selected HMPV-positive healthcare-seeking or tested populations from ED/clinic- and hospital-based studies, the pooled hospitalization proportion was 48%; however, this estimate should be interpreted cautiously because it may reflect testing and selection bias rather than hospitalization risk among all HMPV infections. Among people who are hospitalized, 12% required ICU admission (though possibly subject to small-study effects), 40% needed respiratory support, 8% required mechanical ventilation, and 3% died during hospitalization. These results emphasize that HMPV, while often underrecognized, causes substantial morbidity and mortality across diverse populations and settings.

When benchmarked against other major respiratory pathogens, our pooled ICU admission proportion of 12% falls within the range reported for RSV (10%–20%) and influenza (12%–18%) in people who are hospitalized [5, 10]. Similarly, the overall in-hospital mortality of 3% approaches that of seasonal influenza (4%–6%) and exceeds that of RSV (0.5%–1%) [6, 105]. These findings become even more salient when compared to SARS-CoV-2. While HMPV mortality rate is lower than the 11%–15% observed during the initial waves of the COVID-19 pandemic [106, 107], it closely approaches the 5%–8% in-hospital mortality rates reported for contemporary Omicron-era COVID-19 [108]. These comparisons underscore that HMPV, despite being underrecognized, can produce clinical outcomes of comparable severity to other major vaccine-target respiratory viruses.

Human metapneumovirus contributed to severe outcomes across all age groups, consistent with a broader respiratory virus burden that includes RSV and influenza [9, 109]. In our subgroup analyses, mortality was higher among adults, whereas pediatric hospitalization estimates were influenced by studies enrolling infants and young children. These age-stratified findings should be interpreted cautiously because age categories were broad, mixed-age studies were analyzed separately, and comorbidity-specific estimates were not consistently available. Future studies with finer age strata and standardized comorbidity reporting are needed to clarify population-specific risk patterns. From a public health perspective, these high hospitalization and ICU admission rates highlight a substantial burden on healthcare services, positioning HMPV as a significant contributor to seasonal surges of respiratory illness alongside RSV, influenza, and SARS-CoV-2 [109]. Yet, HMPV remains underrecognized due to overlapping clinical presentations and limited routine testing. Broader implementation of multiplex molecular assays and integration of HMPV into national surveillance platforms are essential for outbreak recognition and burden estimation [109, 110]. Longer-term impacts may also be important, as HMPV has been linked to recurrent wheezing and asthma exacerbations in children [111, 112], while incomplete natural immunity permits reinfection throughout life [56, 113].

While meta-analytic pooling required broad, harmonized age categorizations (<18 and ≥18 years) due to reporting variations in primary source data, the underlying data weights within these strata were primarily driven by the youngest and oldest age cohorts. The pediatric estimates were predominantly derived from studies targeting infants and children under 5 years old, while adult critical care and mortality trends were heavily anchored by cohorts focusing on older adults with chronic health conditions. Consequently, these pooled metrics reflect the substantial clinical burden concentrated within these specific subpopulations and may inform the implementation of age-targeted preventive strategies, surveillance enhancement, and vaccine or monoclonal antibody prioritization frameworks alongside RSV, influenza, and SARS-CoV-2. Encouragingly, vaccine development is advancing, including RSV–HMPV combination candidates in early-phase clinical testing and other HMPV platforms in development [114].

This study has several limitations. First, heterogeneity was substantial across pooled outcomes, reflecting differences in patient populations (age, comorbidities, nutrition, HIV status), study designs (retrospective vs prospective, single- vs multicenter), diagnostic methods (antigen or immunofluorescence vs NAAT), case definitions and severity classifications, admission thresholds and clinical practices, healthcare infrastructure and ICU access, circulating viral subtypes, viral/bacterial coinfections, and reporting quality (incomplete outcomes, inconsistent measurement). Definitions of respiratory support also varied, particularly for CPAP/BiPAP and composite categories combining noninvasive and invasive support; pooled estimates may therefore not represent equivalent interventions. These findings underscore the need for standardized definitions and harmonized reporting in future studies [113]. Hospitalization estimates were particularly susceptible to testing and selection bias because HMPV testing was more likely to be performed in people with more severe illness, in hospital-affiliated settings, or among people meeting specific acute respiratory illness or pneumonia criteria. Differences in healthcare-seeking behavior, testing practices, and admission thresholds across settings may also have contributed to the high heterogeneity observed for hospitalization. Second, because hospitalization analyses excluded community-based surveillance to ensure valid denominators, our estimates apply to people presenting for care and do not capture milder community-managed infections. Furthermore, our reliance on a mixture of studies, some based strictly on people who are hospitalized and others encompassing all people seeking healthcare, may introduce selection bias by pooling populations with different baseline illness severities. Third, data from low- and lower–middle-income countries were less abundant, limiting generalizability in resource-limited settings. Fourth, Egger testing revealed significant small-study effects for ICU admission (*P* = .03), suggesting that the pooled ICU estimate may be modestly inflated, although similar evidence was not observed for other outcomes. Potential publication bias could not be excluded for outcomes with fewer contributing studies [113].

Overall, HMPV is an important cause of severe respiratory disease across age groups [6]. The most immediate implications are to strengthen HMPV surveillance, standardize outcome and denominator reporting, and generate prospective evidence that permits more reliable risk stratification. More granular age- and comorbidity-specific data are needed before these pooled estimates can support age-targeted prevention recommendations or direct comparisons with other respiratory viruses [113].

## Supplementary Material

ofag634_Supplementary_Data

## Supplementary Data

Supplementary materials are available at *Open Forum Infectious Diseases* online. Consisting of data provided by the authors to benefit the reader, the posted materials are not copyedited and are the sole responsibility of the authors, so questions or comments should be addressed to the corresponding author.
