# Supplementary material for: Systematic Review and Meta-Analysis of Clinical Severity and Population-Specific Outcomes of Human Metapneumovirus Infection
Source: Open Forum Infect Dis. 2026 Oct 8;13(10):ofag634. doi: 10.1093/ofid/ofag634 (PMC13693478; doi:10.1093/ofid/ofag634)
Supplement: Supplementary file 1 [file ofag634_supplementary_data.zip › Supplementary_Material_0805_ym.docx]

**eTable 1.** Complete Literature Search Strategies by Database

Search Date: All databases were searched from inception through January 20, 2026.

Language Restrictions: None applied.

| **Database** | **Search Fields & Limits** | **Search Strategy / Query** |
| --- | --- | --- |
| Concept 1: Pathogen | N/A | ("Human metapneumovirus" OR "metapneumovirus" OR "HMPV") |
| Concept 2: Outcomes | N/A | ("severe clinical outcome" OR "severe outcome" OR "severe disease" OR "mortality" OR "death" OR "hospitalization" OR "hospital" OR "hospitalized" OR "inpatient" OR "ICU" OR "mechanical ventilation" OR "fatality") |
| Concept 3: Study Design | N/A | ("cohort" OR "case-control" OR "cross-sectional" OR "prospective" OR "retrospective" OR "longitudinal" OR "observational study" OR "comparative study") |
| Concept 4: Statistics | N/A | ("odds ratio" OR "risk ratio" OR "rate ratio" OR "hazard ratio" OR "incidence rate ratio" OR "multivariate analysis" OR "confidence interval") |
| PubMed | Title/Abstract | Concept 1 AND Concept 2 AND Concept 3 AND Concept 4 |
| Embase | Title/Abstract | Concept 1 AND Concept 2 AND Concept 3 AND Concept 4 |
| Web of Science | Abstract only | Concept 1 AND Concept 2 AND Concept 3 AND Concept 4 |
| Scopus | Title, Abstract, or Keywords | Concept 1 AND Concept 2 AND Concept 3 AND Concept 4 |
| Cochrane Library | Title/Abstract    (Note: Adapted to accommodate platform search term limitations) | Concept 1 AND Concept 2 AND Concept 3 AND Concept 4 |
| medRxiv & bioRxiv | Full Text / Default | "Human metapneumovirus hospital hospitalized clinical outcome severe outcome mortality death study ratio" |
| Google Scholar | Full Text / Default    (Note: First 100 most relevant results screened) | (("Human metapneumovirus" OR "metapneumovirus" OR "HMPV") AND ("severe clinical outcome" OR "severe disease" OR "mortality" OR "death" OR "hospitalization" OR "hospitalized") AND ("cohort" OR "case-control" OR "cross-sectional" OR "prospective" OR "retrospective" OR "longitudinal" OR "observational study" OR "comparative study") AND ("odds ratio" OR "risk ratio" OR "rate ratio" OR "hazard ratio" OR "incidence rate ratio" OR "multivariate analysis" OR "confidence interval")) |

**eTable 2.** Characteristics of Included Studies

| **Paper** | **Country/Region** | **Study Design** | **Setting** | **Population** | **Total Sample (N)** | **HMPV Positive (N)** | **HMPV Positivity (%)** | **Diagnostic Method** |
| --- | --- | --- | --- | --- | --- | --- | --- | --- |
| Aminisani 2025 | New Zealand | Prospective population-based surveillance cohort study | Inpatient | Adults ≥20 years hospitalized with acute respiratory infection (ARI) in Auckland, New Zealand | 21,649 | 735 | 3.4 | RT-PCR, PCR (US CDC real-time RT-PCR; AusDiagnostic PCR protocol) |
| Anderson 2012 | Multinational (US, Canada, Italy, Netherlands, Australia) | Prospective cohort | Inpatient | High-risk children ≤24 months with prematurity, chronic lung disease, and/or congenital heart disease | 1,126 | 99 | 8.8 | NAATs |
| Awad 2020 | Jordan | Prospective cohort | Inpatient | Children aged <5 years diagnosed with ARI | 479 | 33 | 6.9 | NAATs |
| Ayaz 2026 | Bangladesh | Retrospective cohort | Mixed | Patients of any age with RT-PCR confirmed HMPV infection presenting to a tertiary care hospital in Dhaka, Bangladesh | 78 | 78 | 100.0 | RT-PCR |
| Bassiouni 2025 | United States | Prospective cohort | Inpatient | Hospitalized adults with acute respiratory illness (ARI) undergoing respiratory viral testing | 5,432 | 312 | 5.7 | Multiplex PCR |
| Benet 2017 | Multinational (India, Madagascar, Mali, Paraguay) | Prospective cohort | Inpatient | Children 2–60 months hospitalized with radiologically confirmed pneumonia | 405 | 33 | 8.1 | NAATs |
| Berkley 2017 | Kenya | Case-control | Inpatient | Children <13 years with WHO-defined severe or very severe pneumonia | 912 | 23 | 2.5 | NAATs |
| Bhasin 2024 | United States | Cross-sectional | Inpatient | Adults over 18 hospitalized for non-elective, non-surgical reasons | NR | NR | NR | Clinical coding |
| Bhaskar 2025 | United States | Retrospective cohort | Community | Older adults living in VA Community Living Centers with laboratory-confirmed HMPV infection | 178 | 178 | 100.0 | Laboratory-confirmed respiratory viral testing (CLIA-certified VA laboratories; PCR-based panel testing implied) |
| Bhuiyan 2019 | Australia | Case-control | Mixed | Children <18 hospitalized with CAP, age-matched to healthy controls | 460 | 25 | 5.4 | NAATs |
| Boonyaratanakornkit 2020 | Nepal | Prospective cohort | Community | Infants <6 months in rural birth cohort | 3,528 | 187 | 5.3 | NAATs |
| Budge 2014 | Peru | Prospective cohort | Community | Children <3 years in rural highland Peru | 892 | NR | NR | NAATs |
| Bénet 2015 | Mali | Case-control | Mixed | Children <5 years hospitalized with radiologically confirmed pneumonia | 216 | 13 | 6.0 | NAATs |
| Bénet 2017 | Multinational (India, Madagascar, Mali, Paraguay, Cambodia, China, Haiti) | Case-control | Mixed | Children <5 years hospitalized with radiologically confirmed pneumonia | 1,758 | 86 | 4.9 | NAATs |
| Caini 2018 | Ecuador | Cross-sectional | Inpatient | Hospitalized children and adults with SARI | 41,172 | 206 | 0.5 | NAATs |
| Caldera 2025 | United States | Retrospective cohort | Mixed | Children aged 0–5 years tested for respiratory viruses | 9,474 | NR | NR | Multiplex PCR |
| Chen 2020 | China | Prospective cohort | Inpatient | Adults hospitalized with ILI and lab-confirmed Flu, RSV, or HMPV | 594 | 81 | 13.6 | NAATs |
| Chen 2021 | China | Prospective cohort | Inpatient | Adults with PCR-confirmed HMPV, RSV, or hPIV pneumonia | 488 | 137 | 28.1 | NAATs |
| Cheng 2025 | China (Wuhan; 2 hospitals) | Retrospective cohort | Inpatient; hospitalized children at two hospitals | Pediatric hospitalized pneumonia (infectious) | 5,956 | 907 | 15.2 | Multiplex PCR |
| Choi 2025 | United States (Salt Lake City, Utah) | Prospective cohort (RSV) with retrospective comparison cohorts (HMPV) | Inpatient; hospitalized children at two hospitals | Children <5 years hospitalized with laboratory-confirmed viral LRI (RSV/IV/HMPV) | 767 | 68 | 8.9 | Multiplex PCR |
| Chowdhury 2020 | Bangladesh | Case-control | Inpatient | Children aged <5 years with severe malnutrition who met WHO criteria for pneumonia | 694 | 16 | 2.3 | NAATs |
| Do 2016 | Vietnam | Prospective cohort | Inpatient | Children aged <2 years hospitalized with LRTI | 632 | 30 | 4.7 | NAATs |
| Domnich 2025 | Italy (Genoa) | Retrospective cohort | Mixed | Adults aged ≥18 years tested for HMPV by RT-PCR (hospital-based) and adults with ARI/ILI enrolled in sentinel GP surveillance (community-based) | 24,251 | 493 | 2.0 | Multiplex PCR |
| Evelyn 2019 | Colombia | Cross-sectional | Mixed | Children <18 years hospitalized with ARI | 420 | 22 | 5.2 | NAATs |
| Falsey 2021 | Multinational (12 countries: USA, Australia, Argentina, Brazil, Canada, France, Germany, Japan, Malaysia, Mexico, South Korea, South Africa) | Prospective cohort | Inpatient | Adults (≥18 years) hospitalized with ARTI | 709 | 100 | 14.1 | NAATs |
| Foley 2023 | Australia | Retrospective cohort | Inpatient | Children <16 years admitted for respiratory illness | 12,925 | 183 (2021); 65/year average (2017–2019) | NR | NAATs |
| Ghani 2012 | South Africa | Retrospective cohort | Inpatient | Children admitted to PICU with viral RTI | 175 | 12 | 6.9 | NAATs |
| Gil 2017 | Spain | Cross-sectional | Inpatient | Children aged <2 years admitted for LRTI | 451 | 43 | 9.5 | NAATs |
| Goldstein 2025 | USA | Prospective cohort | Mixed | Pediatric (<18 years) | 8,605 | 1,836 | 21.3 | RT-PCR |
| Goriacko 2017 | United States | Retrospective cohort | Inpatient | Children <21 years hospitalized with viral respiratory infection | 1,416 | 66 | 4.7 | NAATs |
| Gray 2006 | United States | Retrospective cohort | Inpatient | A sample of 1,500 archived respiratory specimens collected from October 1, 2001, to May 18, 2004, at the University of Iowa Hospitals and Clinics Clinical Microbiology Laboratory | 1,294 | 34 | 2.6 | NAATs |
| Groome 2015 | South Africa | Prospective cohort | Inpatient | Patients hospitalized with severe acute respiratory illness (SARI) | 17,801 | 712 | 4.0 | NAATs |
| Hahn 2013 | United States | Retrospective cohort | Inpatient | Children aged 0–15 years who were hospitalized, had HMPV identified by direct fluorescent antibody testing in respiratory samples, and had a complete electronic medical record | 238 | 238 | 100.0 | Immunofluorescence |
| Hautala 2021 | Finland | Case-control | Outpatient | Pediatric emergency department patients aged 6 months–6 years | 1,899 | 90 | 4.7 | NAATs |
| Hayashida 2023 | Japan | Retrospective cohort | Inpatient | Patients ≥3 years of age who were hospitalized due to RSV or HMPV infection | 48 | 27 | 56.2 | Antigen detection |
| Hedberg 2022 | Sweden | Retrospective cohort | Inpatient | Hospitalized adults and children aged ≤15 years who tested positive for SARS-CoV-2, influenza A/B, RSV, rhinovirus, enterovirus, parainfluenza virus, HMPV, seasonal coronavirus, adenovirus, or bocavirus | 12,700 | 665 | 5.2 | NAATs |
| Hernández Bautista 2024 | Mexico | Cross-sectional | Mixed | General population | 4,632 | 566 | 12.2 | NAATs |
| Hwang 2017 | South Korea | Retrospective cohort | Inpatient | Hospitalized adult patients | 110 | 110 | 100.0 | NAATs |
| Islam 2026 | Australia | Retrospective cohort | Inpatient | Hospitalized children <5 years with ALRI | 1,081 | 1,081 | 100.0 | Multiplex PCR |
| Jain 2015 | United States | Surveillance | Inpatient | Children aged <18 years hospitalized with pneumonia | 2,222 | 178 | 8.0 | Mixed |
| Jonnalagadda 2017 | Ecuador | Prospective cohort | Inpatient | children with severe pneumonia | 406 | 71 | 17.5 | NAATs |
| Jroundi 2014 | Morocco | Prospective cohort | Inpatient | Children under 5 years old | 689 | 62 | 9.0 | NAATs |
| Kelly 2015 | Botswana | Prospective cohort | Inpatient | Children 1–23 months with pneumonia and were recruited within six hours of presentation to a referral hospital. | 310 | 20 | 6.5 | NAATs |
| Kim 2019 | Korea | Retrospective cohort | Inpatient | Adults with suspected respiratory viral infections | 1,434 | 25 | 1.7 | NAATs |
| Kuang 2024 | China | Retrospective cohort | Inpatient | Pediatric patients with acute respiratory tract illness (ARTI) | 155,165 | 2,524 | 1.6 | NAATs |
| Kumar 2021 | India | Prospective cohort | Community | Adults ≥60 years | 1,403 | 9 | 0.6 | NAATs |
| Lee 2021 | Korea | Retrospective cohort | ICU | Adults aged ≥18 years with community-acquired respiratory viral infection (CA-RVI) | 286 | 26 | 9.1 | NAATs |
| Leece 2023 | Canada | Retrospective cohort | Community | Residents in nursing homes | NR | 1969 per 100 resident-years | NR | NAATs |
| Lemarie 2021 | France | Retrospective cohort | Outpatient | Adult patients who had PCR evidence of a respiratory tract infection (RTI) and were positive for any of the following viruses: influenza A or B virus, RSV, HMPV, and parainfluenza virus (PIV) type 1 (PIV-1) or 3 (PIV-3). | 590 | 90 | 15.3 | NAATs |
| Li 2025 | China | Cross-sectional | Mixed | Children with ARI (<18 years) | 9,834 | 339 | 3.4 | RT-PCR and multiplex PCR |
| Libster 2020 | Argentina | Surveillance | Inpatient | Children aged <2 years | 3,947 | 383 | 9.7 | NAATs |
| Liu 2025 | China | Retrospective cohort | Inpatient | Hospitalized children with severe HMPV and aged ≤17 years | 157 | 157 | 100.0 | PCR |
| Loubet 2020 | France | Retrospective cohort | Inpatient | All adults hospitalized for at least 24 h for ILI | 3,148 | 100 | 3.2 | NAATs |
| Madewell ZJ 2024 | Puerto Rico | Surveillance | Outpatient | Patients of all ages with acute febrile or respiratory illness in Puerto Rico | 43,608 | 862 | 2.0 | NAATs |
| Madhi 2007 | South Africa | RCT | Inpatient | Children hospitalized for RTI | 150 | 19 | 12.7 | Immunofluorescence |
| Marguet 2009 | France | Prospective cohort | Inpatient | Infants <1 year hospitalized with first bronchiolitis episode | 664 | 50 | 7.5 | NAATs |
| McCracken 2014 | Guatemala | Surveillance | Inpatient | Hospitalized patients with acute respiratory infections (all ages) | 1,486 | 97 | 6.5 | NAATs |
| Menezes 2024 | Brazil | Retrospective cohort | Inpatient | Children 0–19 years hospitalized with severe viral LRTI | 78 | 40 | 51.3 | NAATs |
| Mistry 2014 | United States | Prospective cohort | Inpatient | Children 0–19 years presenting to ED with moderate to severe influenza-like illness | 241 | 20 | 8.3 | NAATs |
| Miyakawa 2025 | Multinational (Bangladesh, The Gambia, Kenya, Mali, South Africa, Thailand, Zambia) | Case-control | Mixed | Children 1–59 months hospitalized with severe or very severe pneumonia (HIV-negative) | 8,863 | 382 | 4.3 | NAATs |
| Neu 2012 | United States | Retrospective cohort | Mixed | Medically fragile children and adolescents in a long-term care facility | 150 | 19 | 12.7 | Immunofluorescence |
| Nicolai 2017 | Italy | Retrospective cohort | Inpatient | Full-term children aged <3 years hospitalized with ARTI | 273 | 13 | 4.8 | NAATs |
| Ofman 2020 | Argentina | Cross-sectional | Inpatient | Premature children <2 years rehospitalized with severe LRTI | 664 | 50 | 7.5 | NAATs |
| Ortiz-Hernández 2019 | Mexico | Prospective cohort | Outpatient | Children ≤5 years with influenza-like illness | 1,486 | 97 | 6.5 | NAATs |
| Papenburg 2012 | Quebec City, Canada | Prospective cohort | Inpatient | Children aged <3 years hospitalized in or presenting to a pediatric clinic for acute respiratory infection | 1,039 | 127 | 12.2 | NAATs |
| Phommasone 2022 | Laos | Case-control | Inpatient | Febrile hospitalized patients of any age with acute respiratory symptoms | 394 | 22 | 5.6 | NAATs |
| Pless 2026 | USA | Prospective cohort | Inpatient | Children aged <18 years | 7,982 | 379 | 4.7 | RT-PCR |
| Pott 2025 | Canada | Prospective cohort | Inpatient | Middle-aged and older adults aged ≥50 years hospitalized with acute respiratory illness | 212 | 212 | 100.0 | Multiplex RT-PCR |
| Rayens 2025 | USA (Southern California) | Retrospective cohort | Inpatient | Adults aged ≥18 years hospitalized with ARI | 5,888 | 2,859 | 48.6 | RT-PCR |
| Ricart 2013 | Barcelona, Spain | Prospective cohort | Inpatient | Infants <12 months hospitalized with acute bronchiolitis | 410 | 26 | 6.3 | NAATs |
| Roussy 2014 | Quebec City, Canada | Prospective cohort | Inpatient | Children aged <3 years hospitalized with LRTI | 1,039 | 118 | 11.4 | NAATs |
| Schuster 2015 | Amman, Jordan | Prospective cohort | Inpatient | Children <2 years hospitalized with fever and/or acute respiratory illness | 3,168 | 273 | 8.6 | NAATs |
| Seynaeve 2020 | France | Case series | Mixed | Older adults residing in a long-term care facility | 78 | 40 | 51.3 | NAATs |
| Shi 2023 | China | Retrospective cohort | Mixed | Children aged 0–16 years hospitalized with ARIs | 10,396 | 847 | 8.1 | NAATs |
| Simoes 2025 | USA (Colorado) | Retrospective cohort | Inpatient | Adults aged 50–88 years hospitalized with pneumonia (RSV, HMPV, or influenza) | 10,866 | 1,482 | 13.6 | ICD-10 codes |
| Simoẽs 2025 | USA (Colorado) | Retrospective cohort | Inpatient | Adults aged 50–88 years hospitalized with RSV or HMPV | 6,927 | 2,308 | 33.3 | ICD-10 codes |
| Smyrnaios 2023 | Norway | Case-control | Inpatient | Children <16 years old with radiologically confirmed community-acquired pneumonia | 1,388 | 103 | 7.4 | NAATs |
| Spaeder 2013 | United States | Retrospective cohort | ICU | Children (patients <21 years old) admitted to the ICU with laboratory-confirmed human metapneumovirus (HMPV) infection | 111 | 111 | 100.0 | NAATs |
| Steinberg 2024 | Switzerland | Retrospective cohort | Inpatient | 0–17 years | 110 | 110 | 100.0 | Immunofluorescence |
| Sumino 2018 | United States | Prospective cohort | Inpatient | Children aged ≤5 years with a first episode of HMPV-associated LRTI and no prior asthma or wheezing | 68 | 38 | 55.9 | Immunofluorescence |
| Szilagyi 2016 | United States | Surveillance | Community | General population (>6 months) in Rochester, NY and Marshfield, WI, USA. Random sample, not limited by comorbidity. | 207 | 9 | 4.3 | NAATs |
| Tillekeratne 2019 | Sri Lanka | Prospective cohort | Inpatient | Patients ≥1 year admitted with acute febrile illness (AFI) | 964 | 11 | 1.1 | NAATs |
| Toizumi 2018 | Vietnam | Prospective cohort | Inpatient | Children aged <2 years admitted with acute respiratory infection (ARI) | 1,941 | 47 | 2.4 | NAATs |
| Tong 2016 | Hong Kong, China | Retrospective cohort | ICU | Children admitted to pediatric intensive care unit (PICU) with laboratory-confirmed paramyxovirus infection | 98 | 5 | 5.1 | Immunofluorescence |
| Van Someren Gréve 2018 | Netherlands | Prospective cohort | ICU | Acutely admitted, invasively ventilated adult ICU patients | 1,407 | 7 | 0.5 | NAATs |
| Vos 2021 | Multinational (Spain, Belgium, Poland, UK, Netherlands, Germany, Sweden, Slovakia, Italy, France, Slovenia) | Prospective cohort | Outpatient | Patients aged ≥18 years presenting with acute cough (duration of ≤28 days) and/or suspected LRTI | 2,957 | 121 | 4.1 | NAATs |
| Williams 2004 | United States | Prospective cohort | Mixed | Healthy infants and children under 5 years old; children with acute respiratory tract infection; excluded chronic disease except mild asthma. | 248 | 49 | 19.8 | NAATs |
| Williams 2005 | United States | Prospective cohort | Inpatient | Adults (≥18 years) hospitalized for acute asthma exacerbation | 101 | 7 | 6.9 | NAATs |
| Williams 2010 | United States | Prospective cohort | Inpatient | Children <5 years hospitalized with acute respiratory infection (ARI) or fever | 1,104 | 42 | 3.8 | NAATs |
| Windsor 2022 | United States | Retrospective cohort | Outpatient | Children aged 1 month to 18 years with acute respiratory illness presenting to a pediatric emergency department. | 931 | 36 | 3.9 | NAATs |
| Wolter 2025 | South Africa (KwaZulu-Natal, Mpumalanga, North West provinces) | Case-control | Inpatient | Infants aged <1 year hospitalized with acute respiratory and non-respiratory illness | 1,898 | 43 | 2.3 | Multiplex real-time PCR |
| Wu 2015 | Peru | Prospective cohort | Community | Children aged <3 years in rural high-altitude communities | 892 | 127 | 14.2 | NAATs |
| Yu 2023 | China | Prospective cohort | Mixed | Children (<14 years) hospitalized with acute respiratory tract infection | 5,060 | 1,256 | 24.8 | NAATs |
| Zhou 2019 | China | Prospective cohort | Inpatient | Adults (≥14 years) with community-acquired pneumonia, immunocompetent | 915 | 22 | 2.4 | NAATs |

**eTable 3: Healthcare Utilization in HMPV-Associated Disease**

| **Section** | **Study** | **Population** | **Value (days)** | **Summary measure** |
| --- | --- | --- | --- | --- |
| **HOSPITAL LOS** | **Aminisani 2025** | **HMPV-positive adults** | **4.7 ± 4.1** | **Mean ± SD** |
|  | Aminisani 2025 | HMPV-positive adults | 3 (2–6) | Median (IQR) |
|  | Anderson 2012 | HMPV monoinfection children | 6 (1–28) | Median (range) |
|  | Awad 2020 | HMPV-positive cases | 6.3 (5.2–7.4) | Mean (95% CI) |
|  | Bhasin 2024 | HMPV-positive adults | 12.1 ± 0.3 | Mean ± SD |
|  | Bhuiyan 2019 | HMPV-positive children with CAP | 2 (1–3) | Median (IQR) |
|  | Chen 2020 | HMPV-positive adults | 10 (9–16) | Median (IQR) |
|  | Chen 2021 | HMPV-positive adults | 10 (8–16) | Median (IQR) |
|  | Choi 2025 | HMPV-positive children <5 years | 1.5 (1–2.6) | Median (IQR) |
|  | Choi 2025 | HMPV-positive readmitted children | 2.3 (1.2–3.6) | Median (IQR) |
|  | Do 2016 | HMPV-positive single-infection children | 6 (4–7) | Median (IQR) |
|  | Goldstein 2025 | HMPV-positive inpatient children | 2 (1–4) | Median (IQR) |
|  | Groome 2015 | HMPV-positive children <18 years | 2 (1–5) | Median (IQR) |
|  | Groome 2015 | HMPV-positive adults | 6 (4–8) | Median (IQR) |
|  | Hahn 2013 | HMPV-positive children (all) | 4 (3–6) | Median (IQR) |
|  | Hayashida 2023 | HMPV-positive children ≥3 years | 5 (3–17) | Median (range) |
|  | Hedberg 2022 | HMPV-positive children 0–15 years | 4 (2–6) | Median (IQR) |
|  | Islam 2026 | HMPV monoinfection children (pre-COVID) | 2 (1–3) | Median (IQR) |
|  | Islam 2026 | HMPV monoinfection children (during COVID) | 1 (1–3) | Median (IQR) |
|  | Islam 2026 | HMPV monoinfection children (post-COVID) | 2 (1–3) | Median (IQR) |
|  | Loubet 2020 | HMPV-positive adults | 7 (4–13) | Median (range) |
|  | Madhi 2006 | HMPV-positive children | 3.5 | Mean (range) |
|  | Marguet 2009 | HMPV-positive infants | 4 (3–7.5) | Median (IQR) |
|  | Marguet 2009 | HMPV monoinfection infants | 3.5 (2.5–4.5) | Median (IQR) |
|  | Miyakawa 2025 | HMPV-positive children, severe pneumonia | 4 (3–7) | Median (IQR) |
|  | Neu 2012 | HMPV-positive children ≥5 years | 17 | Mean |
|  | Ortiz-Hernández 2018 | HMPV monoinfection | 3.08 ± 3.03 | Mean ± SD |
|  | Ortiz-Hernández 2018 | HMPV monoinfection | 2 (1–3) | Median (IQR) |
|  | Papenburg 2012 | HMPV-positive children | 3.08 ± 3.03 | Mean ± SD |
|  | Papenburg 2012 | HMPV-positive children | 2 (1–3) | Median (IQR) |
|  | Pott 2025 | HMPV-positive adults ≥50 years | 6 (4–11) | Median (range) |
|  | Rayens 2025 | HMPV-positive adults | 6.3 ± 5.7 | Mean ± SD |
|  | Rayens 2025 | HMPV-positive adults | 5 (4–7) | Median (IQR) |
|  | Schuster 2015 | HMPV-positive children | 4 (3–6) | Median (IQR) |
|  | Seynaeve 2020 | HMPV-positive adults | 7 | Median |
|  | Spaeder 2013 | Severe pediatric cases | 7 (4–18) | Median (IQR) |
|  | Steinberg 2024 | HMPV children (2013/14) | 4.35 (3–5) | Mean (IQR) |
|  | Steinberg 2024 | HMPV children (2021/22) | 5 (2–5) | Mean (IQR) |
|  | Sumino 2013 | HMPV-positive children | 2.8 ± 1.8 | Mean ± SD |
|  | Tong 2016 | HMPV-positive (PICU cohort, total hospital stay) | 24 (6.8–59.3) | Median (IQR) |
| **ICU LOS** |  |  |  |  |
|  | Aminisani 2025 | HMPV-positive adults, ICU | 4.1 ± 3.9 | Mean ± SD |
|  | Aminisani 2025 | HMPV-positive adults, ICU | 2.5 (1.5–6.5) | Median (IQR) |
|  | Anderson 2012 | ICU children | 4 (1–23) | Median (range) |
|  | Evelyn 2019 | HMPV-positive children | 7 (4–14.5) | Median (IQR) |
|  | Hedberg 2022 | HMPV-positive children 0–15 years | 1 (0–4) | Median (IQR) |
|  | Islam 2026 | HMPV monoinfection children, ICU (pre-COVID) | 4.1 (2.0–11.8) | Median (IQR) |
|  | Islam 2026 | HMPV monoinfection children, ICU (during COVID) | 2.4 (1.6–3.5) | Median (IQR) |
|  | Islam 2026 | HMPV monoinfection children, ICU (post-COVID) | 3.7 (2.5–5.9) | Median (IQR) |
|  | Rayens 2025 | HMPV-positive adults, ICU | 6.4 ± 6.5 | Mean ± SD |
|  | Rayens 2025 | HMPV-positive adults, ICU | 4 (2–8) | Median (IQR) |
|  | Spaeder 2013 | Severe pediatric cases | 4 (1–10) | Median (IQR) |
|  | Tong 2016 | HMPV-positive PICU children | 3 (2–25.5) | Median (IQR) |

Abbreviations: CI, confidence interval; HMPV, human metapneumovirus; ICU, intensive care unit; IQR, interquartile range; LTCF, long-term care facility; PERCH, Pneumonia Etiology Research for Child Health; PICU, pediatric ICU; SD, standard deviation.

**eTable 4.** JBI Critical Appraisal Results

| **Study** | **JBI checklist** | **Q1** | **Q2** | **Q3** | **Q4** | **Q5** | **Q6** | **Q7** | **Q8** | **Q9** | **Q10** | **Q11** | **Q12** | **Q13** | **Score (%)** | **Classification*** |
| --- | --- | --- | --- | --- | --- | --- | --- | --- | --- | --- | --- | --- | --- | --- | --- | --- |
| Aminisani 2025 | Surveillance (11) | Y | Y | Y | Y | Y | NA | Y | Y | Y | Y | Y |  |  | 90.9 | Higher quality |
| Anderson 2012 | Cohort (11) | Y | Y | Y | Y | Y | Y | Y | Y | Y | NA | Y |  |  | 90.9 | Higher quality |
| Awad 2020 | Cohort (11) | Y | Y | Y | Y | Y | Y | Y | Y | Y | NA | Y |  |  | 90.9 | Higher quality |
| Ayaz 2026 | Cohort (11) | Y | Y | Y | Y | Y | NA | U | Y | Y | Y | Y |  |  | 81.8 | Higher quality |
| Bassiouni 2025 | Cohort (10) | Y | Y | Y | Y | NA | Y | Y | Y | Y | Y |  |  |  | 90.0 | Higher quality |
| Benet 2017 | Cohort (11) | Y | Y | Y | Y | Y | Y | Y | Y | Y | NA | Y |  |  | 90.9 | Higher quality |
| Berkley 2017 | Case-control (10) | Y | N | Y | Y | Y | Y | Y | NA | NA | Y |  |  |  | 70.0 | Higher quality |
| Bhasin 2024 | Cross-sectional (8) | Y | Y | Y | Y | Y | Y | Y | Y |  |  |  |  |  | 100.0 | Higher quality |
| Bhaskar 2025 | Cohort (10) | Y | Y | Y | Y | NA | Y | Y | Y | Y | Y |  |  |  | 90.0 | Higher quality |
| Bhuiyan 2019 | Case-control (10) | Y | Y | Y | Y | Y | Y | Y | NA | NA | Y |  |  |  | 80.0 | Higher quality |
| Boonyaratanakornkit 2020 | Cohort (11) | Y | Y | Y | N | Y | Y | Y | NA | Y | Y | Y |  |  | 81.8 | Higher quality |
| Budge 2014 | Cohort (11) | Y | Y | Y | Y | Y | Y | Y | Y | Y | N | Y |  |  | 90.9 | Higher quality |
| Bénet 2015 | Case-control (10) | Y | Y | Y | Y | Y | Y | Y | NA | NA | Y |  |  |  | 80.0 | Higher quality |
| Bénet 2017 | Case-control (10) | Y | Y | Y | Y | Y | Y | Y | NA | NA | Y |  |  |  | 80.0 | Higher quality |
| Caini 2018 | Cross-sectional (8) | Y | Y | Y | Y | N | N | Y | Y |  |  |  |  |  | 75.0 | Higher quality |
| Caldera 2025 | Cohort (10) | Y | Y | Y | Y | NA | Y | Y | Y | Y | Y |  |  |  | 90.0 | Higher quality |
| Chen 2020 | Cohort (11) | Y | Y | Y | Y | Y | Y | Y | Y | Y | Y | Y |  |  | 100.0 | Higher quality |
| Chen 2021 | Cohort (11) | Y | Y | Y | Y | Y | Y | Y | NA | Y | Y | Y |  |  | 90.9 | Higher quality |
| Cheng 2025 | Cohort (11) | Y | Y | Y | N | N | U | Y | Y | Y | NA | N |  |  | 54.5 | Lower quality |
| Choi 2025 | Cohort (11) | Y | Y | Y | Y | Y | Y | Y | Y | U | NA | Y |  |  | 81.8 | Higher quality |
| Chowdhury 2020 | Case-control (11) | Y | Y | Y | Y | Y | Y | Y | Y | Y | Y | Y |  |  | 100.0 | Higher quality |
| Do 2016 | Cohort (11) | Y | Y | Y | Y | Y | Y | Y | Y | Y | Y | Y |  |  | 100.0 | Higher quality |
| Domnich 2025 | Cohort (11) | Y | Y | U | Y | Y | Y | Y | Y | N | NA | Y |  |  | 72.7 | Higher quality |
| Evelyn 2019 | Cross-sectional (8) | Y | Y | Y | Y | N | N | Y | Y |  |  |  |  |  | 75.0 | Higher quality |
| Falsey 2021 | Cohort (11) | Y | Y | Y | Y | Y | Y | Y | Y | Y | Y | Y |  |  | 100.0 | Higher quality |
| Foley 2023 | Cohort (11) | Y | Y | Y | NA | NA | Y | Y | Y | Y | N | Y |  |  | 72.7 | Higher quality |
| Ghani 2012 | Cohort (11) | Y | Y | Y | Y | Y | Y | Y | Y | Y | Y | Y |  |  | 100.0 | Higher quality |
| Gil 2017 | Cross-sectional (8) | Y | Y | Y | Y | N | N | Y | Y |  |  |  |  |  | 75.0 | Higher quality |
| Goldstein 2025 | Cohort (11) | Y | Y | Y | Y | Y | Y | Y | Y | U | NA | Y |  |  | 81.8 | Higher quality |
| Goriacko 2017 | Cohort (11) | Y | Y | Y | Y | Y | Y | Y | Y | Y | Y | Y |  |  | 100.0 | Higher quality |
| Gray 2006 | Cohort (11) | NA | NA | Y | Y | Y | NA | Y | NA | NA | NA | Y |  |  | 45.5 | Lower quality |
| Groome 2015 | Cohort (11) | Y | Y | Y | Y | Y | Y | Y | Y | N | NA | Y |  |  | 81.8 | Higher quality |
| Hahn 2013 | Cohort (11) | NA | NA | Y | Y | Y | N | Y | NA | NA | NA | Y |  |  | 45.5 | Lower quality |
| Hautala 2021 | Case-control (10) | Y | Y | Y | Y | Y | Y | Y | Y | U | Y |  |  |  | 90.0 | Higher quality |
| Hayashida 2023 | Cohort (11) | NA | NA | NA | Y | Y | N | Y | Y | Y | NA | Y |  |  | 54.5 | Lower quality |
| Hedberg 2022 | Cohort (11) | NA | NA | Y | Y | Y | N | Y | Y | Y | Y | Y |  |  | 72.7 | Higher quality |
| Hernández Bautista 2024 | Cross-sectional (11) | NA | NA | Y | N | N | NA | Y | N | NA | NA | Y |  |  | 27.3 | Lower quality |
| Hwang 2017 | Cohort (11) | Y | Y | Y | Y | Y | Y | Y | Y | U | U | Y |  |  | 81.8 | Higher quality |
| Islam 2026 | Cohort (11) | Y | Y | Y | Y | Y | Y | Y | Y | Y | NA | Y |  |  | 90.9 | Higher quality |
| Jain 2015 | Surveillance (8) | Y | Y | Y | Y | Y | Y | Y | Y |  |  |  |  |  | 100.0 | Higher quality |
| Jonnalagadda 2017 | Cohort (11) | NA | NA | Y | Y | Y | Y | Y | N | N | NA | Y |  |  | 54.5 | Lower quality |
| Jroundi 2014 | Cohort (11) | Y | Y | Y | Y | Y | Y | Y | Y | U | U | Y |  |  | 81.8 | Higher quality |
| Kelly 2015 | Cohort (11) | Y | Y | Y | Y | Y | Y | Y | Y | Y | Y | Y |  |  | 100.0 | Higher quality |
| Kim 2019 | Cohort (11) | Y | Y | Y | Y | Y | Y | Y | Y | U | U | Y |  |  | 81.8 | Higher quality |
| Kuang 2024 | Cohort (11) | NA | NA | Y | Y | Y | N | Y | Y | Y | NA | Y |  |  | 63.6 | Lower quality |
| Kumar 2021 | Cohort (11) | Y | Y | Y | Y | Y | Y | Y | Y | Y | U | Y |  |  | 90.9 | Higher quality |
| Lee 2021 | Cohort (11) | N | Y | Y | Y | Y | Y | Y | Y | Y | NA | Y |  |  | 81.8 | Higher quality |
| Leece 2023 | Cohort (11) | Y | Y | Y | N | N | Y | Y | Y | U | U | Y |  |  | 63.6 | Lower quality |
| Lemarie 2021 | Cohort (11) | Y | Y | Y | Y | Y | Y | Y | Y | Y | Y | Y |  |  | 100.0 | Higher quality |
| Li 2025 | Cohort (11) | Y | Y | Y | Y | Y | Y | Y | Y | U | NA | Y |  |  | 81.8 | Higher quality |
| Libster 2020 | Surveillance (11) | Y | Y | Y | Y | Y | Y | Y | Y | Y | Y | Y |  |  | 100.0 | Higher quality |
| Liu 2025 | Cohort (11) | Y | Y | Y | Y | Y | Y | Y | Y | U | NA | Y |  |  | 81.8 | Higher quality |
| Loubet 2020 | Cohort (11) | Y | Y | Y | Y | Y | Y | Y | Y | Y | Y | Y |  |  | 100.0 | Higher quality |
| Madewell ZJ 2024 | Surveillance (8) | Y | Y | Y | N | N | Y | Y | Y |  |  |  |  |  | 75.0 | Higher quality |
| Madhi 2007 | RCT (12) | U | Y | U | U | Y | U | U | Y | Y | U | U | Y |  | 41.7 | Lower quality |
| Marguet 2009 | Cohort (11) | Y | Y | Y | U | U | Y | Y | Y | Y | NA | Y |  |  | 72.7 | Higher quality |
| McCracken 2014 | Surveillance (9) | Y | U | Y | Y | Y | Y | Y | Y | NA |  |  |  |  | 77.8 | Higher quality |
| Menezes 2024 | Cohort (11) | Y | Y | Y | Y | Y | U | Y | Y | Y | Y | Y |  |  | 90.9 | Higher quality |
| Mistry 2014 | Cohort (12) | Y | Y | Y | U | NA | Y | Y | Y | Y | Y | NA | Y |  | 75.0 | Higher quality |
| Miyakawa 2025 | Case-control (10) | Y | Y | Y | Y | Y | Y | Y | Y | Y | Y |  |  |  | 100.0 | Higher quality |
| Neu 2012 | Cohort (11) | Y | Y | U | Y | U | Y | Y | Y | Y | NA | Y |  |  | 72.7 | Higher quality |
| Nicolai 2017 | Cohort (11) | Y | Y | Y | Y | U | Y | Y | NA | NA | NA | Y |  |  | 63.6 | Lower quality |
| Ofman 2020 | Cross-sectional (8) | Y | Y | Y | Y | Y | Y | Y | Y |  |  |  |  |  | 100.0 | Higher quality |
| Ortiz-Hernández 2019 | Cross-sectional (8) | Y | Y | Y | Y | Y | Y | Y | Y |  |  |  |  |  | 100.0 | Higher quality |
| Papenburg 2012 | Cohort (11) | Y | Y | Y | Y | Y | Y | Y | Y | Y | Y | Y |  |  | 100.0 | Higher quality |
| Phommasone 2022 | Case-control (10) | Y | Y | Y | Y | Y | Y | Y | Y | U | Y |  |  |  | 90.0 | Higher quality |
| Pless 2026 | Cohort (11) | Y | Y | Y | Y | Y | Y | Y | Y | Y | NA | Y |  |  | 90.9 | Higher quality |
| Pott 2025 | Cohort (11) | Y | Y | Y | Y | Y | Y | Y | Y | Y | NA | Y |  |  | 90.9 | Higher quality |
| Rayens 2025 | Cohort (11) | Y | Y | Y | Y | Y | Y | Y | Y | Y | NA | Y |  |  | 90.9 | Higher quality |
| Ricart 2013 | Cohort (11) | Y | Y | Y | Y | Y | Y | Y | Y | Y | NA | Y |  |  | 90.9 | Higher quality |
| Roussy 2014 | Cohort (11) | Y | Y | Y | N | Y | Y | Y | U | NA | NA | Y |  |  | 63.6 | Lower quality |
| Schuster 2015 | Cohort (11) | Y | Y | Y | Y | Y | Y | Y | Y | Y | Y | Y |  |  | 100.0 | Higher quality |
| Seynaeve 2020 | Case series (10) | Y | N | N | Y | Y | Y | Y | Y | Y | Y |  |  |  | 80.0 | Higher quality |
| Shi 2023 | Cohort (11) | Y | Y | Y | Y | Y | Y | Y | Y | N | N | Y |  |  | 81.8 | Higher quality |
| Simoes 2025 | Cohort (11) | Y | Y | Y | Y | Y | U | Y | Y | Y | NA | Y |  |  | 81.8 | Higher quality |
| Simoẽs 2025 | Cross-sectional (8) | Y | Y | Y | Y | U | N | Y | Y |  |  |  |  |  | 75.0 | Higher quality |
| Smyrnaios 2023 | Case-control (10) | Y | Y | Y | Y | Y | Y | Y | Y | Y | Y |  |  |  | 100.0 | Higher quality |
| Spaeder 2013 | Cohort (11) | Y | Y | Y | Y | Y | Y | Y | Y | Y | NA | Y |  |  | 90.9 | Higher quality |
| Steinberg 2024 | Cohort (11) | Y | Y | Y | N | N | Y | Y | Y | Y | NA | N |  |  | 63.6 | Lower quality |
| Sumino 2018 | Cohort (11) | Y | Y | Y | Y | Y | Y | Y | Y | Y | Y | Y |  |  | 100.0 | Higher quality |
| Szilagyi 2016 | Surveillance (9) | Y | Y | Y | Y | Y | Y | Y | Y | Y |  |  |  |  | 100.0 | Higher quality |
| Tillekeratne 2019 | Cohort (11) | Y | Y | Y | Y | Y | Y | Y | Y | Y | NA | Y |  |  | 90.9 | Higher quality |
| Toizumi 2018 | Cohort (11) | Y | Y | Y | Y | Y | Y | Y | Y | Y | Y | Y |  |  | 100.0 | Higher quality |
| Tong 2016 | Cohort (11) | Y | Y | Y | Y | Y | Y | Y | Y | Y | NA | Y |  |  | 90.9 | Higher quality |
| Van Someren Gréve 2018 | Cohort (11) | Y | Y | Y | Y | Y | Y | Y | Y | Y | U | Y |  |  | 90.9 | Higher quality |
| Vos 2021 | Cohort (11) | Y | Y | Y | Y | Y | Y | Y | Y | Y | Y | Y |  |  | 100.0 | Higher quality |
| Williams 2004 | Cohort (11) | Y | Y | Y | N | N | Y | Y | Y | N | N | Y |  |  | 63.6 | Lower quality |
| Williams 2005 | Cohort (11) | Y | Y | Y | Y | N | Y | Y | Y | Y | N | Y |  |  | 81.8 | Higher quality |
| Williams 2010 | Cohort (9) | Y | Y | Y | Y | Y | Y | Y | Y | Y |  |  |  |  | 100.0 | Higher quality |
| Windsor 2022 | Cohort (11) | Y | Y | Y | Y | Y | Y | Y | Y | N | N | Y |  |  | 81.8 | Higher quality |
| Wolter 2025 | Cohort (11) | Y | Y | Y | Y | Y | Y | Y | Y | Y | NA | Y |  |  | 90.9 | Higher quality |
| Wu 2015 | Cohort (11) | Y | Y | Y | Y | Y | Y | Y | Y | Y | Y | Y |  |  | 100.0 | Higher quality |
| Yu 2023 | Cohort (11) | Y | Y | Y | N | N | U | Y | Y | Y | NA | Y |  |  | 63.6 | Lower quality |
| Zhou 2019 | Cohort (11) | Y | Y | Y | Y | Y | Y | Y | Y | Y | Y | Y |  |  | 100.0 | Higher quality |

Appraisal used the JBI critical appraisal checklist appropriate to each study design. Y, yes; N, no; U, unclear; NA, not applicable.

Score (%) = number of applicable items rated 'yes' divided by the total number of applicable items, multiplied by 100. *For sensitivity-analysis purposes only, studies with ≥70% 'yes' ratings were classified as higher quality and studies with <70% as lower quality. This pragmatic threshold is not a validated JBI risk-of-bias cutoff, and the percentage summary was not used for meta-analytic weighting.

**Cohort (11 items):** Q1, Similar groups/same population; Q2, Exposures measured similarly; Q3, Exposure valid/reliable; Q4, Confounders identified; Q5, Strategies for confounders; Q6, Groups free of outcome at start; Q7, Outcomes measured validly; Q8, Follow-up sufficient; Q9, Follow-up complete/described; Q10, Incomplete follow-up addressed; Q11, Appropriate statistical analysis.

**Case-control (10 items):** Q1, Comparable groups; Q2, Cases/controls matched; Q3, Same identification criteria; Q4, Exposure measured validly; Q5, Exposure measured same way; Q6, Confounders identified; Q7, Strategies for confounders; Q8, Outcomes assessed validly; Q9, Exposure period sufficient; Q10, Appropriate statistical analysis.

**Cross-sectional/Surveillance (8 items):** Q1, Inclusion criteria defined; Q2, Subjects/setting described; Q3, Exposure measured validly; Q4, Objective criteria used; Q5, Confounders identified; Q6, Strategies for confounders; Q7, Outcomes measured validly; Q8, Appropriate statistical analysis.

**Case series (10 items):** Q1, Clear inclusion criteria; Q2, Condition measured reliably; Q3, Valid identification methods; Q4, Consecutive inclusion; Q5, Complete inclusion; Q6, Clear demographics; Q7, Clear clinical information; Q8, Outcomes clearly reported; Q9, Presenting site demographics; Q10, Appropriate statistical analysis.

**RCT (13 items):** Q1, True randomization; Q2, Allocation concealed; Q3, Groups similar at baseline; Q4, Participants blinded; Q5, Treatment deliverers blinded; Q6, Outcome assessors blinded; Q7, Groups treated identically; Q8, Follow-up complete; Q9, Analyzed in assigned groups; Q10, Outcomes measured same way; Q11, Outcomes measured reliably; Q12, Appropriate statistical analysis; Q13, Appropriate trial design.

**Supplementary Figure Legends**

**eFigure 1.** Hospitalization among patients with laboratory-confirmed human metapneumovirus (HMPV) infection, stratified by study setting. Squares indicate study-specific proportions and horizontal lines indicate 95% confidence intervals. Diamonds represent pooled random-effects estimates within each subgroup and overall (GLMM with logit link). The horizontal bar indicates the 95% prediction interval for the overall pooled estimate. Studies are restricted to emergency department/clinic-based and hospital-based settings, excluding community-based surveillance. Abbreviations: CC, case-control; CI, confidence interval; Cross, cross-sectional; HIC, high-income country; HMPV, human metapneumovirus; IF, immunofluorescence; JBI, Joanna Briggs Institute critical appraisal percentage; NAATs, nucleic acid amplification tests; Prosp, prospective; Retro, retrospective; Surv, surveillance; UMIC, upper-middle-income country.

**eFigure 2.** Hospitalization among patients with laboratory-confirmed human metapneumovirus (HMPV) infection, stratified by age group. Squares indicate study-specific proportions and horizontal lines indicate 95% CIs. Diamonds represent pooled random-effects estimates within each subgroup and overall (GLMM with logit link). The horizontal bar indicates the 95% prediction interval for the overall pooled estimate. Age is shown as the minimum–maximum range (m=months; y=years). Abbreviations: CI, confidence interval; HIC, high-income country; HMPV, human metapneumovirus; IF, immunofluorescence; JBI, Joanna Briggs Institute critical appraisal percentage; NAATs, nucleic acid amplification tests; Prosp, prospective; Retro, retrospective; Surv, surveillance; UMIC, upper-middle-income country.

**eFigure 3.** Supplemental respiratory support among patients with laboratory-confirmed human metapneumovirus (HMPV) infection, stratified by study setting. Squares indicate study-specific proportions (with size proportional to study weight), and horizontal lines indicate 95% CIs. Diamonds represent pooled random-effects estimates within each subgroup and overall (GLMM with logit link). The horizontal bar indicates the 95% prediction interval for the overall pooled estimate. Abbreviations: CC, case-control; CI, confidence interval; HIC, high-income country; HMPV, human metapneumovirus; ICU, intensive care unit; IF, immunofluorescence; JBI, Joanna Briggs Institute critical appraisal percentage; LMIC, lower-middle-income country; NAATs, nucleic acid amplification tests; Prosp, prospective; Retro, retrospective; UMIC, upper-middle-income country.

**eFigure 4.** Supplemental respiratory support among patients with laboratory-confirmed human metapneumovirus (HMPV) infection, stratified by age group. Squares indicate study-specific proportions and horizontal lines indicate 95% CIs. Diamonds represent pooled random-effects estimates within each subgroup and overall (GLMM with logit link). The horizontal bar indicates the 95% prediction interval for the overall pooled estimate. Age is shown as reported by each study (minimum–maximum range or median [IQR]; m=months; y=years). Abbreviations: CC, case-control; CI, confidence interval; HIC, high-income country; HMPV, human metapneumovirus; IF, immunofluorescence; JBI, Joanna Briggs Institute critical appraisal percentage; LMIC, lower-middle-income country; NAATs, nucleic acid amplification tests; Prosp, prospective; Retro, retrospective; UMIC, upper-middle-income country.

**eFigure 5.** ICU admission among patients with laboratory-confirmed human metapneumovirus (HMPV) infection, stratified by age group. Squares indicate study-specific proportions and horizontal lines indicate 95% CIs. Diamonds represent pooled random-effects estimates within each subgroup and overall (GLMM with logit link). The horizontal bar indicates the 95% prediction interval for the overall pooled estimate. Age is shown as reported by each study (minimum–maximum range or median [IQR]; m=months; y=years). Abbreviations: CC, case-control; CI, confidence interval; Cross, cross-sectional; HIC, high-income country; HMPV, human metapneumovirus; ICU, intensive care unit; IF, immunofluorescence; JBI, Joanna Briggs Institute critical appraisal percentage; LMIC, lower-middle-income country; NAATs, nucleic acid amplification tests; Prosp, prospective; Retro, retrospective; Surv, surveillance; UMIC, upper-middle-income country.

**eFigure 6.** Mechanical ventilation among patients with laboratory-confirmed human metapneumovirus (HMPV) infection, stratified by study setting. Squares indicate study-specific proportions and horizontal lines indicate 95% CIs. Diamonds represent pooled random-effects estimates within each subgroup and overall (GLMM with logit link). The horizontal bar indicates the 95% prediction interval for the overall pooled estimate. Abbreviations: CI, confidence interval; Cross, cross-sectional; HIC, high-income country; HMPV, human metapneumovirus; ICU, intensive care unit; IF, immunofluorescence; JBI, Joanna Briggs Institute critical appraisal percentage; LMIC, lower-middle-income country; NAATs, nucleic acid amplification tests; PCR, polymerase chain reaction; Prosp, prospective; Retro, retrospective; UMIC, upper-middle-income country.

**eFigure 7.** Mechanical ventilation among patients with laboratory-confirmed human metapneumovirus (HMPV) infection, stratified by age group. Squares indicate study-specific proportions and horizontal lines indicate 95% CIs. Diamonds represent pooled random-effects estimates within each subgroup and overall (GLMM with logit link). The horizontal bar indicates the 95% prediction interval for the overall pooled estimate. Age is shown as reported by each study (minimum–maximum range or median [IQR]; m=months; y=years). Abbreviations: CI, confidence interval; Cross, cross-sectional; HIC, high-income country; HMPV, human metapneumovirus; IF, immunofluorescence; JBI, Joanna Briggs Institute critical appraisal percentage; LMIC, lower-middle-income country; NAATs, nucleic acid amplification tests; Prosp, prospective; Retro, retrospective; UMIC, upper-middle-income country.

**eFigure 8.** In-hospital mortality among patients with laboratory-confirmed human metapneumovirus (HMPV) infection, stratified by age group. Squares indicate study-specific proportions and horizontal lines indicate 95% CIs. Diamonds represent pooled random-effects estimates within each subgroup and overall (GLMM with logit link). The horizontal bar indicates the 95% prediction interval for the overall pooled estimate. Age is shown as reported by each study (minimum–maximum range or median [IQR]; m=months; y=years). Abbreviations: CC, case-control; CI, confidence interval; Cross, cross-sectional; HIC, high-income country; HMPV, human metapneumovirus; IF, immunofluorescence; JBI, Joanna Briggs Institute critical appraisal percentage; LIC, low-income country; LMIC, lower-middle-income country; NAATs, nucleic acid amplification tests; Prosp, prospective; Retro, retrospective; UMIC, upper-middle-income country.

**eFigure 9.** Funnel plot for assessment of small-study effects in studies reporting in-hospital mortality among HMPV-positive patients (n=29 studies). Each circle represents an individual study. The vertical line represents the pooled effect estimate, and the diagonal dotted lines indicate approximate 95% funnel limits. Egger's test: P=.518.

**eFigure 10.** Funnel plot for assessment of small-study effects in studies reporting ICU admission among HMPV-positive patients (n=26 studies). Each circle represents an individual study. The vertical line represents the pooled effect estimate, and the diagonal dotted lines indicate approximate 95% funnel limits. Egger's test: P=.031.

**eFigure 11.** Funnel plot for assessment of small-study effects in studies reporting mechanical ventilation among HMPV-positive patients (n=16 studies). Each circle represents an individual study. The vertical line represents the pooled effect estimate, and the diagonal dotted lines indicate approximate 95% funnel limits. Egger's test: P=.952.

**eFigure 12.** Funnel plot for assessment of small-study effects in studies reporting supplemental respiratory support among HMPV-positive patients (n=18 studies). Each circle represents an individual study. The vertical line represents the pooled effect estimate, and the diagonal dotted lines indicate approximate 95% funnel limits. Egger's test: P=.545.
